# Epidemiology of Idiopathic Pulmonary Fibrosis in Northern Italy

**DOI:** 10.1371/journal.pone.0147072

**Published:** 2016-02-03

**Authors:** Sergio Harari, Fabiana Madotto, Antonella Caminati, Sara Conti, Giancarlo Cesana

**Affiliations:** 1 Unità Operativa, di Pneumologia e Terapia Semi-Intensiva Respiratoria, Servizio di Fisiopatologia Respiratoria ed Emodinamica Polmonare, Ospedale San Giuseppe-MultiMedica IRCCS, Milan, Italy; 2 Research Centre on Public Health, Department of Medicine and Surgery, University of Milano-Bicocca, Milan, Italy; National Institute of Health, ITALY

## Abstract

**Background:**

Idiopathic pulmonary fibrosis (IPF) is the most common and severe form of idiopathic interstitial pneumonia. Despite its clinical relevance, few studies have examined the epidemiology of IPF and temporal variation in disease incidence and prevalence. Aim of the study was to investigate the prevalence, incidence and trends of IPF in Lombardy, a region with nearly 10 million inhabitants, during 2005–2010.

**Methods:**

For the identification of IPF patients, we used healthcare administrative databases of Lombardy Healthcare System and adopted three algorithms: generic, broad and narrow case definition (GCD, BCD, NCD). IPF cases were identified according to diagnoses reported in inpatient and outpatient claims occurred during 2000–2010. We estimated age- and sex-adjusted annual prevalence and incidence rates from 2005 to 2010, thus allowing for a 5-year washout period.

**Results:**

The mean annual incidence rate was estimated at 2.3 and 5.3 per 100,000 person-years using NCD and GCD, respectively. IPF incidence was higher among males, and increased with age. Trend remained stable over the years. The estimated annual prevalence rate was 35.5, 22.4, and 12.6 per 100,000 person-years using GCD, BCD and NCD, respectively, and increased with age. Moreover, we observed a positive trend over the years. Using BCD and NCD, prevalence was higher among males.

**Conclusions:**

The results of this study, which is one of the largest population-based survey ever conducted according to strict criteria, indicated that prevalence of IPF increased across the years while incidence remained stable, thus suggesting that survival with IPF has improved.

## Introduction

Idiopathic pulmonary fibrosis (IPF) is the most common and severe form of idiopathic interstitial pneumonia [1]. It is a progressive disease with a variable clinical course and is associated with extremely poor prognosis [2–4]. IPF is characterized by a short survival from diagnosis (3–5 years) and a high fatality rate [5]. Respiratory failure is the most common cause of death; however, other causes of death, such heart failure, bronchogenic carcinoma, ischemic heart disease, infection and pulmonary embolism, are frequently concurrent [6,7]. The disease pathogenesis is not fully understood; however, abnormal wound healing processes and molecular alterations involved in aging and inflammatory related processes appear to be involved [8,9].

One of the major challenges in epidemiologic studies of such a rare disease has been the difficulty of recruiting a sufficient number of patients [10]. Few large-scale studies have specifically focused on epidemiological investigations [9, 11–17]. and the true incidence and prevalence of IPF are not well established. Difficulties encountered in establishing the epidemiology of IPF may also be due to the lack of uniform definition of IPF in older studies and differences in diagnostic criteria, study population and design [18,19]. Thus, incidence and prevalence of IPF vary across the studies. Recent studies indicated that the incidence and mortality of IPF appear to be on the rise [12,18,20,21]; however, data on incidence trends are not always in agreement [22].

Epidemiology of IPF in Italy has been poorly investigated. In two studies, the prevalence of IPF has been evaluated analysing data collected in the Italian Registry of Diffuse Infiltrative Pulmonary Diseases, a multicentre prospective registry established in 1998 [23,24]. However, only the percentage of IPF diagnosis among patients affected by interstitial lung disease (ILD) was evaluated. Moreover, this registry was created using data obtained from those centres that voluntarily accepted to adhere to the project and, therefore, from those physicians who spontaneously provided information on number and clinical characteristics of patients with ILDs. It is likely that the use of this registry might have resulted in an underestimation of IPF cases.

In a recent study carried out in the Lazio region (which has about 6 million inhabitants), the epidemiology of IPF was investigated using hospital admissions and mortality databases of the regional health system [13]. The annual incidence and prevalence in this large Central-Southern Italian region were estimated at 7.5 and 25.6 per 100,000 person-years, respectively; however Italian trends in incidence and prevalence of IPF are unknown.

The aim of this study was to evaluate the epidemiology of IPF in Lombardy—the most populous region of Italy—between 2005 and 2010, by analysing the health care administrative databases.

## Materials and Methods

To investigate the epidemiological characteristics of IPF in Lombardy, a region with nearly 10 million inhabitants (accounting for 16.3% of the Italian population), we performed an observational retrospective analysis of healthcare administrative databases of the Health Regional System.

Since the Italian Health Care System provides universal coverage, each region is responsible for collecting and organizing data on health care services provided to patients; moreover, each region is charged with storing all such information in databases for administrative purposes. In order to facilitate the use of these databases for scientific purposes, since 2000 in Lombardy all data have been inserted in a new data warehouse, named DENALI. DENALI is endowed with a probabilistic record linkage capable of ameliorating the match of anonymized data of different datasets belonging to the same individual [25–27].

For each resident in Lombardy, DENALI contains the following data: demographic characteristics and vital status; hospitalization data, including up to six discharge diagnoses encoded according to the International Classification of Diseases, ninth Revision, Clinical Modification, 2002 edition (ICD-9-CM) and up to six procedures; description of each outpatient visit, with the indication of diagnoses (encoded according to ICD-9-CM) or procedures performed during that visit.

For our analysis, we first applied a case definition that we called “generic case definition” (GCD) and that identified as IPF cases all individuals with at least one hospitalization with diagnosis of IPF or at least one outpatient visit with diagnosis of IPF (ICD-9-CM code 516.3) during the period from 1st January 2000 to 31st December 2010. For the identification of cases as incident or prevalent through claim analysis, the date of the first event (index event) of each patient was used as a proxy for the timing of disease onset. In addition to GDC, we used the two case definitions suggested by Raghu et al. [28], namely the “broad case definition” (BCD) and the “narrow case definition” (NCD), with some adjustments in order to adapt them to our context. In detail, for each subject satisfying GCD we extracted from DENALI all claims occurred before or after the index event. We then applied the BCD: we defined IPF cases those patients that satisfied the GCD and had no claims (inpatient or outpatient) with a diagnosis code for any other type of ILDs (Table 1) on or after the date of the last claim with IPF diagnosis. Finally, we applied the NCD: we defined IPF cases those patients that satisfied the BCD and had one or more claim with a procedure code for surgical lung biopsy, transbronchial lung biopsy or computed tomography of the thorax (Table 1), on or before the date of the last claim with a diagnosis code for IPF.

**Table 1:** Criteria applied in general, broad and narrow case definitions for IPF.

Case definition	Criteria
General	Subjects with at least one hospital admission or outpatient visit with IPF diagnosis (ICD-9-CM code 516.3)
Broad	Subjects with:
	■ at least one hospital admission or outpatient visit with IPF diagnosis (ICD-9-CM code 516.3)
	■ no hospital admission or outpatient visit with ILDs diagnosis (ICD-9-CM codes 135, 237.7, 272.7, 277.3, 277.8, 446.21, 446.4, 495, 500, 501, 502, 503, 504, 505, 506.4, 508.1, 508.8, 515, 516.0, 516.1, 516.2, 516.8, 516.9, 517.2, 517.8, 518.3, 555, 710.0, 710.1, 710.2, 710.3, 710.4, 714.81, 720.0, 759.5) on or after date of last IPF diagnosis (ICD-9-CM code 516.3)
Narrow	Subjects with:
	■ at least one hospital admission or outpatient visit with IPF diagnosis (ICD-9-CM code 516.3)
	■ no hospital admission or outpatient visit with ILDs diagnosis (ICD-9-CM codes 135, 237.7, 272.7, 277.3, 277.8, 446.21, 446.4, 495, 500, 501, 502, 503, 504, 505, 506.4, 508.1, 508.8, 515, 516.0, 516.1, 516.2, 516.8, 516.9, 517.2, 517.8, 518.3, 555, 710.0, 710.1, 710.2, 710.3, 710.4, 714.81, 720.0, 759.5) on or after date of last IPF diagnosis (ICD-9-CM code 516.3)
	■ at least one surgical lung biopsy (ICD-9-CM codes 33.28), transbronchial lung biopsy (ICD-9-CM codes 33.27) or computed tomography of the thorax (ICD-9-CM codes 87.41) performed during an hospitalization or outpatient visit, on or before date of last IPF diagnosis (ICD-9-CM code 516.3)

IPF, idiopathic pulmonary fibrosis; ICD-9-CM, international classification of diseases, 9^th^ revision; ILD, interstitial lung disease.

Since DENALI doesn’t include information about health services provided prior to 2000, and the median survival of patients is 3–5 years after the onset of IPF [1,6], the use of DENALI data from 2000 to 2004 might result in an underestimation of the number of prevalent cases and, at the same time, an overestimation of the number of incident cases until 2004. To ensure that the index event is a good proxy for the onset of IPF, information about health system accesses should be documented for at least five years before the onset itself. For these reasons, we only analysed the period 2005–2010, and incident IPF patients were identified among patients who had been covered by the Lombardy health care system for at least 5 years before the index event (washout period).

Using the three case definitions (GCD, BCD, NCD), we assessed the overall prevalence and incidence rates of IPF as well as prevalence and incidence rates stratified by gender and age (<55, 55–59, 60–64, 65–69, 70–74, 75–79, 80–84, and ≥85 years). Finally, we evaluated temporal trends by computing yearly prevalence and incidence rates standardized by age and gender, using the population living in Lombardy at January 1st 2010 as reference [29].

We assumed a Poisson distribution of the rates and computed 95% confidence interval (95% CI) based on Normal approximation [30]. In order to test for age-related and temporal linear trends, for each case definition we computed the count of expected incident (or prevalent) cases in the reference population stratified by gender, age classes and calendar year, and then we used these counts as dependent variable in a Poisson regression model with the following structure:

$$\text{log E}\left({\text{Y}}_{\text{i}}\right)=\text{log}\;N_{i}+\beta_{0}+\beta_{1}{\text{I}}_{\text{Males},\text{i}}+\beta_{2}{\text{Age}}_{\text{i}}+\beta_{3}{\text{Year}}_{\text{i}}$$ (1)

where *Y*_*i*_ is the expected count in the *i*-th stratum; *N*_*i*_ is person-years at risk in the *i*-th stratum; *I*_*Males*,*i*_ is an indicator variable that assumes value 1 when the gender is male in the *i*-th stratum; *Age*_*i*_ is the age class of the *i*-th stratum and *Year*_*i*_ is the calendar year of the *i*-th stratum. A significant Wald’s test for *β*_2_ and *β*_3_ indicated respectively an age-related and a temporal linear trend in the dependent variable.

For all statistical tests, a pre-specified two-sided α of 0.05 was regarded as significant.

The analyses were performed using SAS software, version 9.2 (SAS Institute, Cary, NC, USA) and R, version 3.1.1 (R Project for Statistical Computing, http://www.R-project.org).

## Results

### Study population

Using the DENALI data warehouse, we found that a total of 11,558 hospital admissions with diagnosis of IPF and 5,117 outpatient visits with an IPF diagnosis occurred over the years 2000–2010. Limiting the analysis to 2005–2010, the number of subjects with IPF identified through the hospital discharge diagnoses alone were 4,872 using the GCD, 3,255 using the BCD, and were 2,094 using the NCD; however, since we also searched for IPF diagnosis in outpatient visits, sample sizes raised, and a total of 5,441 (+11.7%), 3,573 (+9.8%), and 2,097 (+0.1%) IPF patients were identified using the GCD, BCD and NCD, respectively (data not shown).

### Prevalence

The number of IPF cases identifies as prevalent in Lombardy during 2005–2010 is reported in Table 2: the results are illustrated according to the three IPF case definitions, and stratified by gender and age groups. Based on the GCD, BCD and NCD, the number of prevalent cases of IPF was 5,441 (53.8% of whom were male), 3,573 (male: 57.2%) and 2,097 (male: 56.9%), respectively. About 70% of patients were aged 65 and older, regardless of the case definition.

**Table 2:** Demographic characteristics of prevalent and incident cases of IPF from 2005 to 2010 in Lombardy by case definition.

	Prevalent cases			Incident cases		
	GCD	BCD	NCD	GCD	BCD	NCD
	N (%)	N (%)	N (%)	N (%)	N (%)	N (%)
**Total**	5,441	3,573	2,097	2,951	2,093	1,309
**Gender**						
Male	2,929 (53.83)	2,042 (57.15)	1,193 (56.89)	1,674 (56.73)	1,252 (59.82)	772 (58.98)
Female	2,512 (46.17)	1,531 (42.85)	904 (43.11)	1,277 (43.27)	841 (40.18)	537 (41.02)
**Age group (years)**						
<55	860 (15.81)	515 (14.41)	306 (14.59)	361 (12.23)	242 (11.56)	155 (11.84)
55–59	429 (7.88)	259 (7.25)	161 (7.68)	200 (6.78)	132 (6.31)	83 (6.34)
60–64	530 (9.74)	338 (9.46)	217 (10.35)	293 (9.93)	201 (9.60)	142 (10.85)
65–69	737 (13.55)	482 (13.49)	302 (14.40)	400 (13.55)	288 (13.76)	185 (14.13)
70–74	870 (15.99)	578 (16.18)	348 (16.60)	500 (16.94)	346 (16.53)	216 (16.50)
75–79	929 (17.07)	607 (16.99)	354 (16.88)	544 (18.43)	382 (18.25)	238 (18.18)
80–84	720 (13.23)	516 (14.44)	278 (13.26)	416 (14.10)	315 (15.05)	186 (14.21)
85+	366 (6.73)	278 (7.78)	131 (6.25)	237 (8.03)	187 (8.93)	104 (7.94)

GCD, generic case definition; BCD, broad case definition; NCD, narrow case definition.

Using to the GCD, the estimated average annual prevalence rate (per 100,000 person-years) was 35.51 (95% CI: 35.02–36.00), with no significant differences between genders (Table 3).

**Table 3:** Estimates of the mean annual standardized IPF prevalence rates (per 100,000 person-years) in Lombardy during the period 2005–2010 by gender, age, and case definition.

	GCD	BCD	NCD
**Adjusted rates per 100,000 person-years (95% CI)**			
Overall	35.51 (35.02–36.00)	22.39 (21.99–22.78)	12.55 (12.26–12.84)
Male	35.19 (34.51–35.87)	23.64 (23.08–24.20)	13.23 (12.82–13.65)
Female	35.84 (35.13–36.55)	21.07 (20.52–21.62)	11.84 (11.43–12.25)
Age classes			
<55	9.77 (9.46–10.08)	5.67 (5.44–5.91)	3.22 (3.04–3.39)
55–59	45.34 (43.18–47.51)	27.09 (25.42–28.76)	16.98 (15.66–18.30)
60–64	60.45 (57.85–63.05)	35.47 (33.48–37.46)	20.90 (19.37–22.43)
65–69	84.79 (81.66–87.93)	53.94 (51.44–56.44)	32.21 (30.27–34.14)
70–74	106.36 (102.60–110.13)	68.83 (65.80–71.85)	39.47 (37.17–41.76)
75–79	133.37 (128.66–138.08)	84.43 (80.69–88.17)	46.40 (43.62–49.17)
80–84	143.71 (137.89–149.53)	97.14 (92.36–101.92)	51.28 (47.81–54.75)
85+	99.20 (93.66–104.74)	72.72 (67.98–77.46)	32.54 (29.37–35.71)
**Unadjusted rates per 100,000 person-years (95% CI)**			
**Male**			
<55	10.29 (9.84–10.75)	4.96 (4.65–5.28)	3.51 (3.25–3.79)
55–59	46.65 (43.63–49.83)	23.27 (21.11–25.59)	19.38 (17.45–21.46)
60–64	63.57 (59.91–67.38)	28.87 (26.34–31.57)	25.26 (22.97–27.71)
65–69	84.07 (79.83–88.48)	51.28 (47.79–54.96)	33.47 (30.81–36.29)
70–74	100.67 (95.80–105.72)	69.17 (64.71–73.86)	37.97 (35.00–41.12)
75–79	118.03 (112.37–123.91)	91.82 (85.80–98.15)	42.43 (39.07–46.01)
80–84	108.53 (102.41–114.92)	131.18 (121.95–140.92)	41.88 (38.11–45.92)
85+	53.87 (49.31–58.75)	158.36 (144.90–172.73)	19.68 (16.96–22.71)
**Female**			
<55	9.28 (8.86–9.71)	6.42 (6.07–6.79)	2.93 (2.70–3.18)
55–59	43.98 (40.99–47.12)	30.75 (28.31–33.35)	14.48 (12.79–16.33)
60–64	57.13 (53.56–60.88)	41.68 (38.74–44.80)	16.26 (14.39–18.32)
65–69	85.60 (81.07–90.31)	56.32 (52.85–59.94)	30.80 (28.10–33.67)
70–74	113.12 (107.40–119.07)	68.53 (64.53–72.72)	41.24 (37.81–44.90)
75–79	154.52 (146.68–162.67)	79.07 (74.45–83.91)	51.86 (47.36–56.67)
80–84	205.43 (193.84–217.53)	77.75 (72.58–83.18)	67.78 (61.19–74.88)
85+	224.63 (208.54–241.63)	41.77 (37.76–46.09)	68.13 (59.41–77.78)

GCD, generic case definition; BCD, broad case definition; NCD, narrow case definition; 95%CI, 95% confidence interval.

The prevalence of diagnosed IPF increased with increasing age, rising from 9.77 for people aged less than 55 years (95% CI: 9.46–10.08) to 143.71 for people aged 80–84 years (95% CI: 137.89–149.53). However, prevalence dropped to 99.20 (95% CI: 93.66–104.74) for people aged 85 years and older.

Using the BCD, the estimated mean annual prevalence rate (per 100,000 person-years) was 22.39 (95% CI: 21.99–22.78), and was significantly higher in males (23.64; 95% CI: 23.08–24.20) than females (21.07; 95% CI: 20.52–21.62). The age-stratified analyses yielded similar results to those obtained using the GCD, with the lowest mean annual prevalence being in the youngest group of age (5.67; 95% CI: 5.44–5.91) and the highest among people aged 80–84 years (97.14; 95% CI: 92.36–101.92); then, mean annual prevalence decreased to 72.72 (95% CI: 67.98–77.46) for people aged 85 years and older.

Finally, the average annual prevalence rate (per 100,000 person-years) as estimated according to the NCD was 12.55 (95% CI: 12.26–12.84). As observed using the BCD, prevalence was higher in males (13.23; 95% CI: 12.82–13.65) than in females (11.84; 95% CI: 11.43–12.25); moreover, the age-stratified analyses showed a pattern that was similar to that obtained using the GCD and BCD.

The trends observed in age classes were confirmed by the Poisson regression models (Table 4) for all case definitions: the estimated β for age class was always significantly positive.

**Table 4:** Results of Poisson regression models on expected prevalence rates in the reference population (population of Lombardy at January 1^st^ 2010).

	GCD			BCD			NCD		
	β	SE	p-value^a^	β	SE	p-value^a^	β	SE	p-value^a^
Calendar year	0.0298	0.0040	<0.0001	0.0499	0.0051	<0.0001	0.0580	0.0068	<0.0001
Age class	0.3790	0.0026	<0.0001	0.3942	0.0033	<0.0001	0.3767	0.0044	<0.0001
Gender									
Male	0.2543	0.0140	<0.0001	0.1328	0.0177	<0.0001	0.1198	0.0236	<0.0001
Female	ref.			ref.			ref.		

GCD, Generic Case Definition; BCD, Broad Case Definition; NCD, Narrow Case Definition; SE, Standard Error.

^a^ Wald’s test.

**Fig 1:**
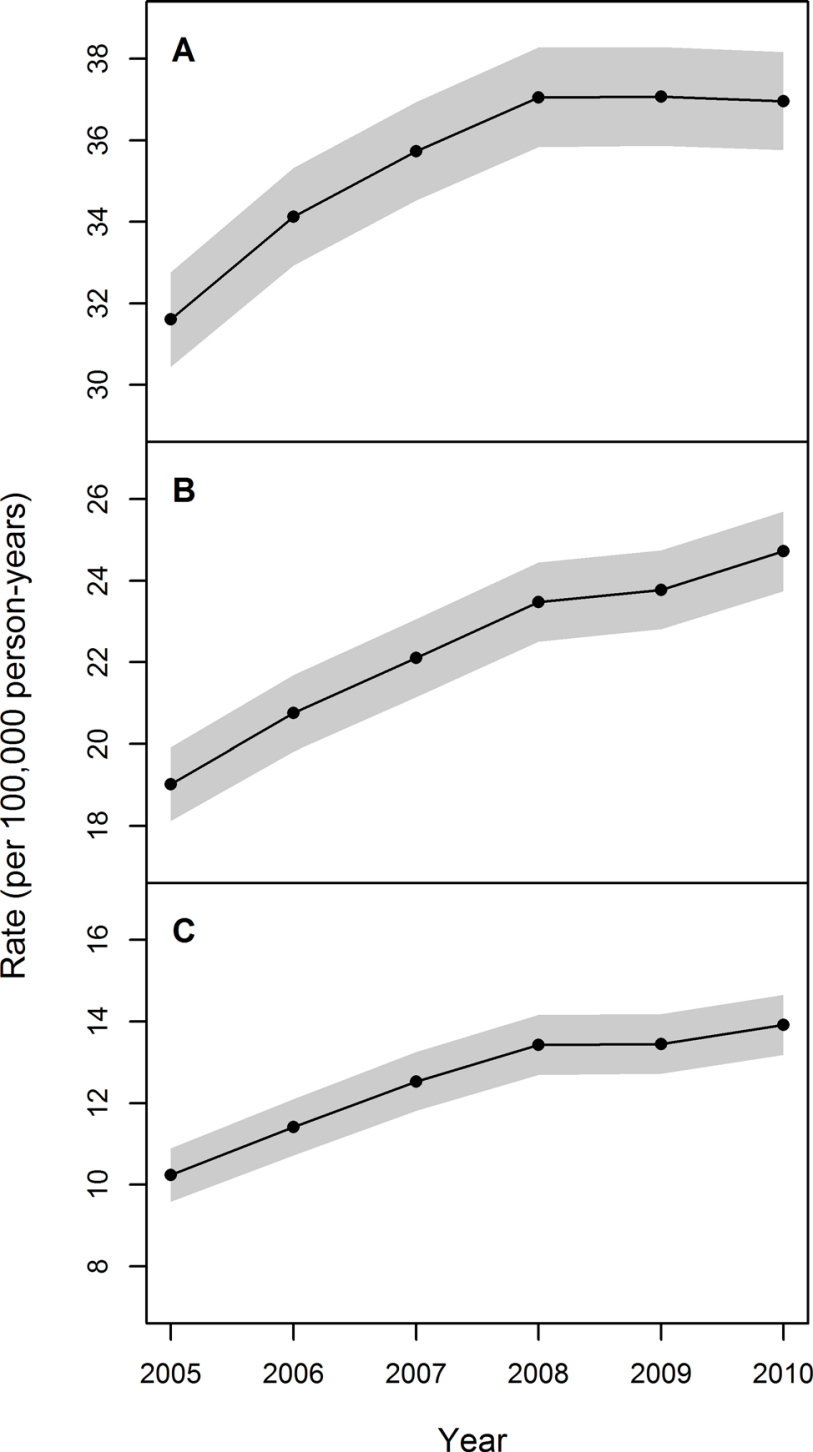
Trend in age-sex standardized annual prevalence rates (per 100,000 person-years) of IPF in Lombardy during 2005–2010, by case definition. (A) General case definition. (B) Broad case definition. (C) Narrow case definition. Grey bands indicate 95% confidence intervals.

The analysis of the temporal trend revealed that the annual standardized prevalence rates increased from 2005 to 2008, and seemed to stabilize thereafter, regardless of the case definition (Fig 1). However, the stabilization was more evident when the analysis was performed using the GCD. The Poisson model reported in Table 4, detected an overall growing trend related to calendar year.

### Incidence

From 2005 to 2010, a total of 2,951, 2,093 and 1,309 new cases of IPF were registered in Lombardy using the GCD, BCD and NCD, respectively. Results are illustrated in Table 2. About 60% of new cases occurred in males, regardless of the case definition. The majority of incident cases (63% of the total) occurred in subjects aged 65 to 84 years. Age distribution of incident cases did not significantly differ among the three case definitions.

Using the GCD, the estimated annual incidence rate (per 100,000 person-years) was 5.25 (95% CI: 5.06–5.44), and was significantly higher among males (6.18; 95% CI: 5.88–6.48) than females (4.37; 95% CI: 4.13–4.61) (Table 5).

**Table 5:** Estimates of the mean annual standardized IPF incidence rates (per 100,000 person-years) in Lombardy during the period 2005–2010 by gender, age, and case definition.

	GCD	BCD	NCD
**Adjusted rates per 100,000 person-years (95% CI)**			
Overall	5.25 (5.06–5.44)	3.74 (3.58–3.90)	2.33 (2.20–2.46)
Male	6.18 (5.88–6.48)	4.63 (4.37–4.89)	2.85 (2.65–3.05)
Female	4.37 (4.13–4.61)	2.88 (2.69–3.08)	1.84 (1.68–2.00)
Age classes			
<55	0.92 (0.82–1.01)	0.62 (0.54–0.69)	0.39 (0.33–0.46)
55–59	5.36 (4.62–6.10)	3.54 (2.93–4.14)	2.22 (1.75–2.70)
60–64	8.51 (7.54–9.49)	5.84 (5.03–6.65)	4.13 (3.45–4.80)
65–69	12.08 (10.90–13.27)	8.70 (7.70–9.70)	5.59 (4.78–6.39)
70–74	17.40 (15.88–18.93)	12.06 (10.79–13.34)	7.53 (6.52–8.53)
75–79	23.69 (21.70–25.68)	16.68 (15.01–18.36)	10.40 (9.08–11.72)
80–84	25.59 (23.13–28.06)	19.40 (17.26–21.55)	11.45 (9.81–13.10)
85+	18.91 (16.50–21.31)	14.93 (12.79–17.07)	8.29 (6.69–9.88)
**Unadjusted rates per 100,000 person-years (95% CI)**			
**Male**			
<55	0.98 (0.85–1.13)	0.70 (0.59–0.82)	0.42 (0.34–0.52)
55–59	5.68 (4.64–6.88)	4.15 (3.27–5.20)	2.57 (1.89–3.41)
60–64	10.98 (9.45–12.69)	7.80 (6.52–9.26)	5.46 (4.40–6.71)
65–69	15.81 (13.90–17.91)	11.65 (10.02–13.47)	7.49 (6.20–8.98)
70–74	21.67 (19.20–24.36)	16.33 (14.20–18.68)	9.83 (8.19–11.69)
75–79	33.80 (30.18–37.72)	25.61 (22.48–29.06)	16.05 (13.59–18.82)
80–84	40.98 (35.90–46.58)	31.87 (27.41–36.86)	18.74 (15.36–22.64)
85+	34.53 (28.41–41.59)	28.00 (22.52–34.42)	14.62 (10.74–19.44)
**Female**			
<55	0.86 (0.73–1.00)	0.53 (0.43–0.65)	0.36 (0.28–0.46)
55–59	5.05 (4.09–6.17)	2.95 (2.23–3.83)	1.90 (1.33–2.62)
60–64	6.19 (5.09–7.46)	3.99 (3.12–5.04)	2.87 (2.14–3.77)
65–69	8.74 (7.41–10.24)	6.05 (4.96–7.32)	3.88 (3.02–4.92)
70–74	13.81 (12.04–15.76)	8.47 (7.10–10.03)	5.59 (4.49–6.87)
75–79	16.37 (14.30–18.64)	10.21 (8.59–12.04)	6.30 (5.05–7.77)
80–84	16.83 (14.47–19.46)	12.30 (10.29–14.57)	7.30 (5.78–9.10)
85+	13.26 (11.04–15.78)	10.21 (8.28–12.45)	6.00 (4.54–7.77)

GCD, generic case definition; BCD, broad case definition; NCD, narrow case definition; 95%CI, 95% confidence interval.

Similarly to prevalence rate, the incidence rate increased with increasing age, with the lowest value being in the youngest age group: the rate (per 100,000 persons-years) rose from 0.92 among people aged less than 55 years (CI%: 0.82–1.01) to 25.59 among people aged 80–84 years (95% CI: 23.13–28.06), and then decreased to 18.91 (95% CI: 16.50–21.31) for the oldest population (85+ age group).

Using the BCD, the overall annual incidence rate (100,000 person-years) was 3.74 (95% CI: 3.58–3.90). The rate was significantly higher among males (4.63; 95% CI: 4.37–4.89). The age-stratified analysis showed a pattern that was similar to that observed using the GCD, although the point estimates were lower: the lowest incidence rate, estimated for people aged less than 55 years, was 0.62 (95% CI: 0.54–0.69), and the highest rate (which was estimated for people aged 80 to 84 years) was 19.40 (95% CI: 17.26–21.55).

Using the NCD, the mean annual incidence rate (per 100,000 person-years) was estimated at 2.33 (95% CI: 2.20–2.46), with higher values in males (2.85; 95% CI: 2.65–3.05) than females (1.84; 95% CI: 1.68–2.00). The age-stratified analysis revealed a pattern that was similar to that observed using the GCD and BCD: the incidence rate rose from 0.39 among people younger than 55 years of age (95% CI: 0.33–0.46) to 11.45 among people aged 80–84 years (95% CI: 9.81–13.10), and then dropped for subjects aged 85 years and older.

For all case definitions, Poisson regression models (Table 6) confirmed the significance of the trends observed for age classes: the estimated β for age class was always significantly positive.

**Table 6:** Results of Poisson regression models on expected incidence rates in the reference population (population of Lombardy at January 1^st^ 2010).

	GCD			BCD			NCD		
	β	SE	p-value^a^	β	SE	p-value^a^	β	SE	p-value^a^
**Overall model**									
Calendar year	-0.0683	0.0105	<0.0001	-0.0270	0.0124	0.0298	-0.0310	0.0158	0.0492
Age class	0.4598	0.0069	<0.0001	0.4782	0.0083	<0.0001	0.4628	0.0104	<0.0001
Gender									
Male	0.6552	0.0367	<0.0001	0.8026	0.0440	<0.0001	0.7467	0.0556	<0.0001
Female	ref.			ref.			ref.		
**Model excluding year 2009 **									
Calendar year	-0.0560	0.0114	<0.0001	-0.0044	0.0132	0.7411	-0.0004	0.0166	0.9814
Age class	0.4527	0.0074	<0.0001	0.4699	0.0088	<0.0001	0.4531	0.0110	<0.0001
Gender									
Male	0.6547	0.0394	<0.0001	0.8147	0.0473	<0.0001	0.7878	0.0594	<0.0001
Female	ref.			ref.			ref.		

GCD, Generic Case Definition; BCD, Broad Case Definition; NCD, Narrow Case Definition; SE, Standard Error.

^a^ Wald’s test.

**Fig 2:**
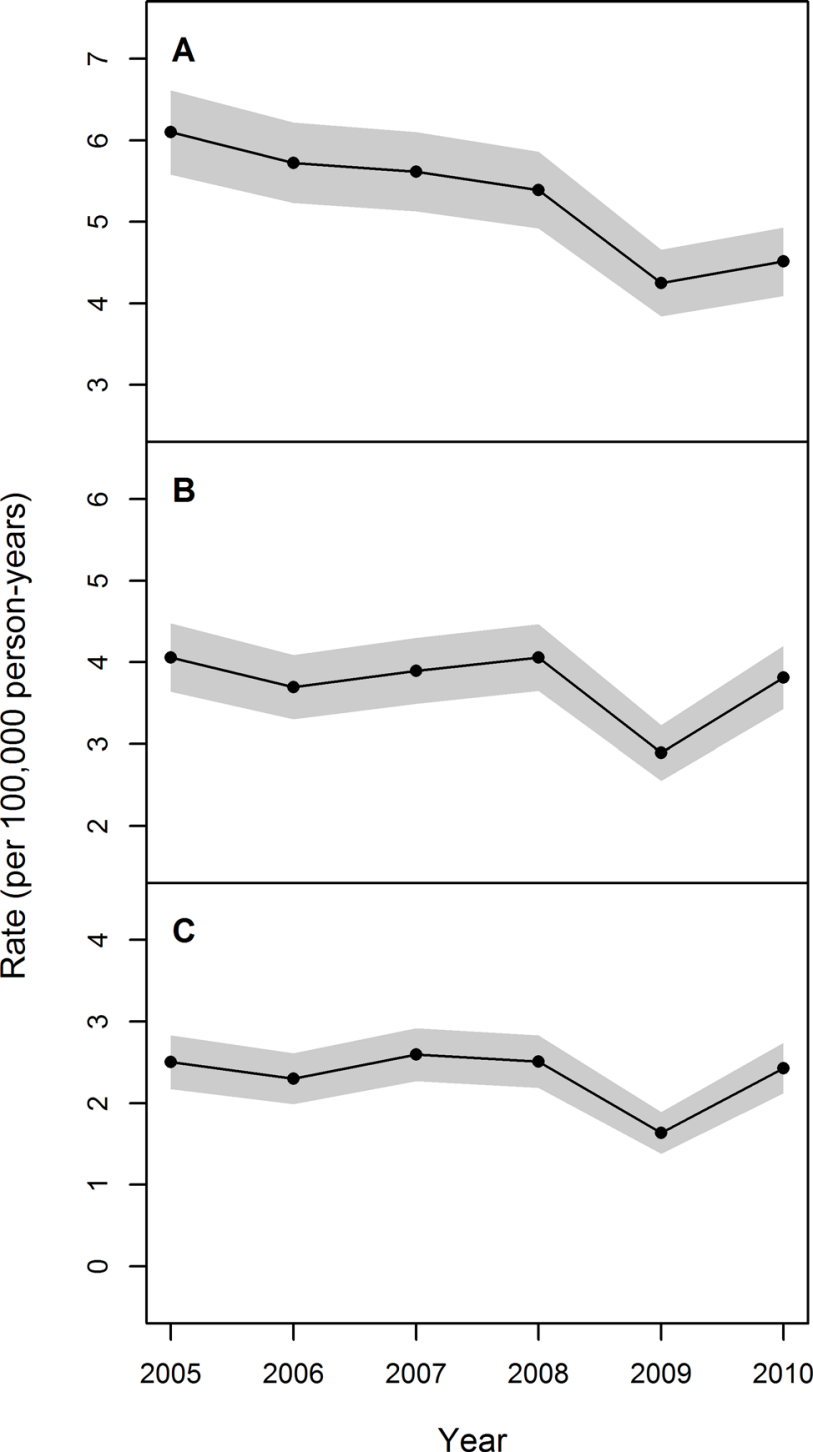
Trend in age-sex standardized annual incidence rates (per 100,000 person-years) of IPF in Lombardy during 2005–2010 by case definition. (A) General case definition. (B) Broad case definition. (C) Narrow case definition. Grey bands indicate 95% confidence intervals.

The analysis of the temporal trend during the period 2005–2010 revealed that, using the GCD, the estimated annual incidence rates slightly decreased, with a more consistent decline over the last two years (Fig 2). However, the analysis of temporal trend using the BCD and NCD revealed that the annual incidence rates were stable during the study period, with the only exception of the year 2009, when a sudden decrease was observed. The overall Poisson regression model detected a significant negative trend for all case definition (Table 6). However, such trend was not confirmed for BCD and NCD when the model excluded 2009 data.

## Discussion

In our study we used healthcare administrative databases to evaluate epidemiology of IPF in Lombardy, which, with nearly 10 million inhabitants in 2013, is the most populous region of Italy. As highlighted in a recent review [19], the analysis of health care databases is one of the most common methodologies used to identify cases of IPF. This approach provides information from a large population without the expenditure required by the creation of a national registry; moreover, it is critical for accruing a sufficient sample size for epidemiological studies for rare diseases such IPF.

In our study, the estimated mean annual incidence rate of IPF varied between 2.3 and 5.3 per 100,000 person-years, and the estimated prevalence rate varied between 12.6 and 35.5 per 100,000 person-years, depending on the case definition used to identify IPF patients.

The comparison between our results and findings from other studies is hampered by the variety of IPF case definitions and by differences in study population, time period of analysis, and geographic locations. In the US, two different research groups studied the incidence and prevalence of IPF by analyzing medical claims databases [28,31]; however, the broad and narrow definitions that were used in those studies included slightly different criteria. Raghu et al. [28] estimated the incidence rate to be 6.8 and 16.3 per 100,000 person-years in the year 2000 using their narrow and broad definition, respectively; in the study of Fernandez-Perez et al. [31], which focused on the epidemiology of IPF during the period 1997–2005, the estimated incidence rate was 8.8 per 100,000 person-years based on their narrow definition, and was 17.4 per 100,000 person-years based on their broad definition. The overall prevalence of IPF estimated by Raghu et al. [28] was 14.0 and 42.7 per 100,000 person-years using their narrow and broad definition, respectively, while the prevalence estimated by Fernandez-Perez et al. [31] was 27.9 and 63.0 per 100,000 person-years by their narrow and broad definition criteria, respectively. It should be noted that only residents aged 50 years and older were evaluated in the Fernandez-Perez et al. study [31].

In Japan, only one study has been conducted to investigate the epidemiology of IPF [17], and the results suggested that the estimated prevalence and incidence in 2008 appear to be much lower (10.0 and 2.2 per 100,000 person-years, respectively) than those observed in the US population.

In general, IPF estimates of prevalence and incidence rates appear to be lower in Europe relative to those reported in the US population [18,20,22]. In particular, studies that investigated the IPF epidemiology during the first years of the 21st century–which, therefore, are comparable to our study—found that the estimated mean annual incidence rate (per 100,000 person-years) ranged from 0.9 in Greece [32] to 7.4 in UK [33]; the estimated prevalence rate was only evaluated in Greece, and was 3.4 [32]. Recently, the study of Agabiti et al. [13] focused on epidemiology of IPF in the adult population of a region of Central-Southern Italy using hospital admission records: findings indicated that the estimated annual incidence rate over the period 2005–2009 varied between 7.5 and 9.3 (per 100,000 person-years), and that the estimated IPF prevalence in 2009 varied between 25.6 and 31.6 (per 100,000 person-years), depending on the case definition. These results show a higher incidence and a lower prevalence rate than ours. These differences might be due to different demographic characteristics of the analysed populations, or to environmental factors, for example Lazio and Lombardy differ for climate as well as concentration and sources of environmental pollution, or they might be even due to differences in the patient management, as each region of Italy has its own Healthcare System that is to some extent autonomous. However, we can’t rule out that, they might also originate from differences in study design: first, our estimates refer to the whole population, while those from Agabiti are related to people aged 18 or older; second, the criteria used in the study of Agabiti et al. were similar to the GCD criteria used in our study, but did not include outpatient claims; finally, different washout periods were used to identify IPF cases as incident or prevalent.

Our findings suggested that prevalence and incidence of IPF in Lombardy might be similar to those estimated in other European countries, and thus lower compared to those observed in the United States; however, the use of different IPF case definitions and differences in subject selection criteria might invalidate a direct comparison of findings among countries [18,19].

Our results are consistent with several previous surveys finding that incidence and prevalence of IPF are higher among men, and increase with increasing age [11,17,28,31,33–35]. Interestingly, we observed that both prevalence and incidence of IPF significantly decreased in the oldest age group (aged 85 years and older). A similar pattern of incidence and prevalence rates was observed in the study conducted in UK by Gribbin et al. [35], and might be related to clinical complexities inherent in elderly patients.

In our study, we not only assessed the prevalence and incidence of IPF but also provided information about temporal trend. To our knowledge, only four studies have focused on this aspect [11,31,33,35]. Two of these studies investigated temporal trends before 2005 [31,35]. The other two studies indicated that incidence rates remained stable in the UK [33] and the US [11] since 2005 and that IPF prevalence in the US has increased annually in recent years [10]. Results from our study are in line with such findings: the analysis of temporal trend revealed that the annual incidence rates estimated using the BCD and NCD were stable in Lombardy during the study period, while IPF prevalence (per 100,000 person-years) increased from 19.0 in 2005 to 24.7 in 2010 using BCD, and from 10.2 in 2005 to 13.9 in 2010 using NCD (Fig 1). This increase might be attributable to a gain in survival of patients developing IPF during the last years as a result of recent advances in the diagnosis and management of the disease and in particular of comorbidities [11]. However, it should be noted that we observed a significant drop in incidence rate during 2009. Such anomaly might be the consequence of a change in legislation establishing administrative and management protocols in Lombardy: indeed, in this period it was established that some health care services that were previously provided in day-hospitals would be provided in outpatient settings. The adaptation to the new legislation might have resulted in a temporary reduction in the number of hospitalizations in 2009, which, however, ended before the following year. Such adaptation problems may also account for the results on prevalence trend: the increase in prevalence observed from 2005 to 2007 flattened between 2008 and 2009; afterwards, prevalence rate continued to increase. In order to assess the influence of the drop on the overall mean annual rates, we computed rates excluding the year 2009 and we obtained slightly higher incidence rates: 5.46 (95% CI: 5.24–5.67), 3.91 (95%CI: 3.73–4.09) and 2.47 (95%CI: 2.33–2.62) respectively for GCD, BCD and NCD; and we estimated minimally lower prevalence rates: 35.18 (95%CI: 34.64–35.72), 22.10 (95%CI: 21.67–22.52), 12.37 (95%CI: 12.05–12.68). It should be noted that the differences with the estimates reported in Tables 3 and 5 are not significant because the confidence intervals always overlap. The same conclusion applies to age or gender stratified rates (results not shown). Therefore, we can conclude that including 2009 in the analysis does not lead to bias in the estimated rates.

Our study was carried out using health care administrative databases and, therefore, it has some limitations, as already suggested by Raghu et al [11,28]. First, patients with IPF were identified based on their access to health care services: as a matter of fact, it was impossible to determine the exact timing of the onset of IPF and, therefore, the date of the first encounter with the inpatient or outpatient healthcare system facility coinciding with the first recorded medical diagnostic claim of IPF was used as a proxy for the timing of disease onset. Second, the accuracy of the reported diagnoses in the hospital admissions databases was unknown as we could not integrate in the study a clinical review of each medical chart or of a representative sample of them. In the study of Agabiti et al. [13], it was found that a number of revised hospital charts carrying the ICD9-CM 515 code (post inflammatory pulmonary fibrosis), 516.8 code (other specified alveolar and parietoalveolar pneumonopathies) and 516.9 code (unspecified alveolar and parietoalveolar pneumonopathies) were eventually redefined as “IPF confident” cases. Therefore, some extent of misdiagnosis or misreporting of IPF cases might occur, thus resulting in an underestimation of IPF cases [13]. Moreover, the sensitivity of the ICD-9-CM code 516.3 in identifying IPF cases might be low [13], as this code includes some extremely rare conditions such as the alveolar capillary block and Hamman-Rich syndrome [11]; furthermore, some other ILDs might be wrongly coded as 516.3 without appropriate health examinations. We partially overcame this latter limitation by recognizing patients using the broad and narrow case definitions, which consider patients’ medical history before and after the presumed IPF onset and, therefore, exclude those patients for whom such types of miscoding might have occurred. Nevertheless, as we observed that patients who were likely to be misclassified as having IPF received a diagnosis of ILD other than IPF within two years after the presumed onset, a certain extent of misclassification might have persisted in 2009 and 2010.

From a methodological point of view, we based our case definitions on the most rigorous available criteria that were suitable for the analysis of the administrative data [11,28]; in addition, we included outpatient claims. Compared to the study of Agabiti et al. [13], this approach had the advantage of allowing identification of patients who had never been admitted to hospital because of IPF (and accounted for about 10% of IPF cases, as observed using the general and broad case definitions). Furthermore, the identification of IPF cases as prevalent or incident was based on criteria that were much stricter than those used in the above-mentioned studies [11,28]: indeed, we used a longer washout period (5-years) in order to minimize the probability of misclassification of prevalent as incident IPF case.

Despite the above-mentioned limitations, we believe that health care administrative databases are useful tools to investigate the epidemiology of a rare disease like IPF. This approach allowed us to select a sample of population that was larger than those evaluated in most of the published studies. Indeed, this was one of the biggest sample ever considered, together with studies conducted in UK [33,35]: to date, the largest sample in US was composed of roughly 3.7 million inhabitants [11,28], which accounted for about 37% of the population selected for our study. Besides, our study investigated for the first time trends in IPF incidence and prevalence in an Italian region.

Moreover, thanks to the universal health care coverage and to the DENALI data warehouse, which traces a complete medical history of each resident in Lombardy by merging data of different datasets belonging to the same individual, we could investigate the prevalence and incidence of IPF in an unselected population, without restrictions related to age [11,13,31], adherence to some health plan (e.g. Medicare) [11,28], or voluntary recruitment [17,32].

In conclusion, our results on IPF prevalence and incidence are in line with those reported in other epidemiological studies conducted in Italy and Europe, and incidence and prevalence trends are in agreement with those reported in European and American studies. The convergence of findings from different epidemiological studies of IPF—some of which were also performed using administrative databases—is a very important achievement, considering all the challenges that are faced in investigating epidemiology of rare diseases (e.g. diagnostic issues and difficulties of study design). Future studies are warranted to validate the accuracy of the diagnostic codes used to identify IPF cases in large databases, as well as to investigate different target populations, living in areas with different geographical, social and environmental characteristics.
